# Temporal and Isoform-Specific Expression of *CTBP2* Is Evolutionarily Conserved Between the Developing Chick and Human Retina

**DOI:** 10.3389/fnmol.2021.773356

**Published:** 2022-01-13

**Authors:** Elizabeth Gage, Devansh Agarwal, Calvin Chenault, Kameron Washington-Brown, Sarah Szvetecz, Nusrat Jahan, Zixiao Wang, Melissa K. Jones, Donald J. Zack, Ray A. Enke, Karl J. Wahlin

**Affiliations:** ^1^Department of Biology, James Madison University, Harrisonburg, VA, United States; ^2^Department of Bioengineering, University of California, San Diego, La Jolla, CA, United States; ^3^Shiley Eye Institute, Viterbi Family Department of Ophthalmology, University of California, San Diego, La Jolla, CA, United States; ^4^Department of Mathematics & Statistics, James Madison University, Harrisonburg, VA, United States; ^5^The Center for Genome & Metagenome Studies, James Madison University, Harrisonburg, VA, United States; ^6^Wilmer Eye Institute, Johns Hopkins University School of Medicine, Baltimore, MD, United States; ^7^Department of Genetic Medicine, Johns Hopkins University School of Medicine, Baltimore, MD, United States; ^8^Department of Molecular Biology and Genetics, Johns Hopkins University School of Medicine, Baltimore, MD, United States

**Keywords:** chicken, human, retina, development, RIBEYE, CTBP2, organoid

## Abstract

Complex transcriptional gene regulation allows for multifaceted isoform production during retinogenesis, and novel isoforms transcribed from a single locus can have unlimited potential to code for diverse proteins with different functions. In this study, we explored the CTBP2/RIBEYE gene locus and its unique repertoire of transcripts that are conserved among vertebrates. We studied the transcriptional coregulator (CTBP2) and ribbon synapse-specific structural protein (RIBEYE) in the chicken retina by performing comprehensive histochemical and sequencing analyses to pinpoint cell and developmental stage-specific expression of CTBP2/RIBEYE in the developing chicken retina. We demonstrated that CTBP2 is widely expressed in retinal progenitors beginning in early retinogenesis but becomes limited to GABAergic amacrine cells in the mature retina. Inversely, RIBEYE is initially epigenetically silenced in progenitors and later expressed in photoreceptor and bipolar cells where they localize to ribbon synapses. Finally, we compared CTBP2/RIBEYE regulation in the developing human retina using a pluripotent stem cell derived retinal organoid culture system. These analyses demonstrate that similar regulation of the CTBP2/RIBEYE locus during chick and human retinal development is regulated by different members of the K50 homeodomain transcription factor family.

## Introduction

Visual perception is initiated in the retina when photons of light are absorbed by rod and cone photoreceptor (PR) neurons. Visual stimuli received at PRs are transmitted to bipolar cells (BCs) followed by retinal ganglion cells (RGC) that project to higher-order neurons in the brain. While PRs, BCs, and RGCs provide vertical signaling within the retina, horizontal cells (HCs) and amacrine cells (ACs) provide lateral inhibition for contrast sensitivity and high visual acuity. PR and BC ribbon synapses are easily distinguished from conventional synapses by their electron-dense horseshoe-shaped structures that lie perpendicular to the synaptic membrane (Morgans, 2000a, b). These synapses are somewhat rare within the CNS in that they are restricted to cochlear hair cells of the inner ear, pinealocytes of the pineal gland, and PRs and BCs of the retina. While rod PRs typically have one to two ribbons (Carter-Dawson and LaVail, 1979; Migdale et al., 2003; Sterling and Matthews, 2005), cone PRs have up to 30 (Ahnelt et al., 1990). Unlike conventional synapses that have wide active zones, synaptic contacts beneath ribbons are uniquely channeled for the tonic release of glutamate using a large complex of proteins, including the scaffold proteins RIBEYE (CTBP2), PICCOLO, and BASOON which link actin filaments with synaptic vesicle release (Takamori et al., 2006).

In mammals, two distinct *CTBP2* isoforms arise from alternative promoter usage at the *CTBP2* locus. “RIBEYE” is the name given to the longer synaptic ribbon-associated isoform which is composed of a synapse-specific A-domain and a shorter C-terminal B-domain that is identical to a second short nuclear “CTBP2” isoform, except for a 20 amino acid nuclear localization domain that is specific to the short isoform (Schmitz et al., 2000). Although the role of RIBEYE as a key structural protein in ribbon synapses is well known, the role of CTBP2 is less well understood (Schmitz et al., 2000). The CTBP2 protein is a transcriptional coactivator in a wide variety of cells (He et al., 2021; Kainoh et al., 2021; Meijer et al., 2021) and its importance is highlighted by the fact that *CTBP2* null mice do not survive past embryonic day 10 (Hildebrand and Soriano, 2002). Although its role in the retina is not well understood, CTBP2 does bind with many proteins that influence transcription, including histone deacetylases (HDACs). In the inner ear, SOX6 influences cell fate by recruitment of CTBP2 and HDAC1 to modulate gene expression (Iguchi et al., 2007). Thus, insights into other systems might give some clues about its role in the eye.

The chicken embryo (*Gallus gallus*) is a classic cone dominant model system for studying retinogenesis. In the chick, like many other species, synaptic development in the inner retina precedes a second wave of synaptogenesis in the outer retina. While we previously documented that synaptic ribbon assembly in the chick retina bears some resemblance to other species (Wahlin et al., 2008, 2010), the molecular composition of chicken retina synapses is still incomplete. Using immunohistochemistry (IHC) with antibodies against CTBP2 to label ribbons in the mature chick retina (Wahlin et al., 2010), we observed that rods, cones, and BCs each displayed RIBEYE at the inner and outer plexiform layers (IPL/OPL). To further explore when and where synaptic ribbons formed in the retina during development, we also studied a developmental series of chick retina sections which provided us detailed information on the temporal dynamics of CTBP2 expression during retinogenesis. While protein expression filled in some knowledge gaps, we noted that the *CTBP2* genomic locus was still incomplete and lacked RIBEYE even in the most recent galGal6 assembly. To identify and sequence *RIBEYE*, we used 5’RACE and RNA-seq. This gave detailed sequence information for both *CTBP2* short and long isoforms. We then determined the positional identity of CTBP2+ cells using single cell chicken RNAseq (scRNA-seq) data mined from publicly available datasets. Finally, we analyzed human retinal organoids during retinal development using RNAseq which provided evidence that *CTBP2* and *RIBEYE* show similar expression patterns and are evolutionarily conserved with the chick. These findings provide a foundational understanding of CTBP2 and RIBEYE in this important model organism and demonstrate mechanistic similarities to human retinal development.

## Materials and Methods

### Animals and Human Tissue

Animal tissue collection was carried out in accordance with animal protocols approved by the Animal Care and Use Committee at James Madison University. White Leghorn chick embryos (*Gallus domesticus*) from B&E Eggs (York Springs, PA) and Cobb/Hubbard F1 hybrid from George’s Hatchery (Harrisonburg, VA) were incubated in a rocking chamber held at 38°C and 50–60% humidity until specified incubation/post-hatch days. All uses of human tissues were carried out in accordance with the Institutional Review Board at JMU. Informed consent from the donor next-of-kin was collected prior to adult human tissue collection. Left and right pairs of whole globe human donor eyes were curated from the National Disease Research Interchange.

### Cells

PSCs were used with authorization from the UC San Diego Institutional Review Board Committee. Line IMR90.4 iPSCs were obtained from WiCell (Madison, Wisconsin). Cells were routinely tested for mycoplasma by PCR (Drexler and Uphoff, 2002). Pluripotency of cells was evaluated with antibodies for NANOG, OCT4, SOX2, and SSEA4.

### PSC Maintenance

Stem cells were maintained antibiotic free on 1% (vol/vol) Matrigel-GFR (#354230; BD Biosciences) coated dishes at 37°C under hypoxic conditions (10% CO_2_/5%O_2_) in mTeSR1 (Stem Cell Technologies; Ludwig et al., 2006; Yao et al., 2006; Chen et al., 2011; Wahlin et al., 2017). Cells were passaged every 4–6 days, with Accutase (#A6964; Sigma) for 8–10 min, dissociated to single cells, quenched with mTeSR1 plus 5 μM (−) blebbistatin (B; #B0560; Sigma), pelleted at 80× *g* for 5 min, resuspended in mTeSR1+B and plated at 5,000 cells per 35 mm dish (Walker et al., 2010). After 48 h, cells were fed without B.

### Human Retinal Organoid Differentiation

Human pluripotent stem cells (hPSCs) and hPSC-derived retinal organoids were cultured as previously described (Wahlin et al., 2017). Briefly, stem cells were passaged with Accutase for 12 min and 1,000 cells in 50 μl’s of mTeSR1+B were seeded per well into a polystyrene 96-well U-bottom plate (#650180; Greiner). Over the first 4 days, aggregates were transitioned to neural induction medium (BE6.2-NIM) by adding 50 μl BE6.2 + 2% MG on day 1 and 50 μl BE6.2 + 1% MG each day thereafter. On D4–8 a 50% medium exchange (100 μl) was performed daily and every other day thereafter. NIM also contained 3 μM of the WNT antagonist (IWR-1-endo; #681669 EMD Millipore) from D1 to D6. For hypoxia experiments, feeding occurred in ambient air for approximately 5 min and returned to hypoxia for growth. Organoids were grown in BE6.2+300nM Smoothened agonist (SAG; #566660; EMD Millipore) from D8 to D14 and then LTR+SAG from D14 to D18. For longer term experiments (e.g., RNAseq) we used sharpened tungsten needles to excise optic vesicles from D10 to 12 as previously described (Wahlin et al., 2017). Organoids were maintained in suspension in LTR at low density (<24–36/ 10 cm untreated 10 cm polystyrene Petri dishes) and fed every 2–3 days. Poorly defined vesicles were periodically removed. To increase survival and differentiation, 500 nM all-trans retinoic acid (ATRA; #R2625; Sigma) was added to LTR from D20 and 10 μM DAPT from D28 to D42.

### Immunoblotting and Immunohistochemistry (IHC)

**Figure 1:**
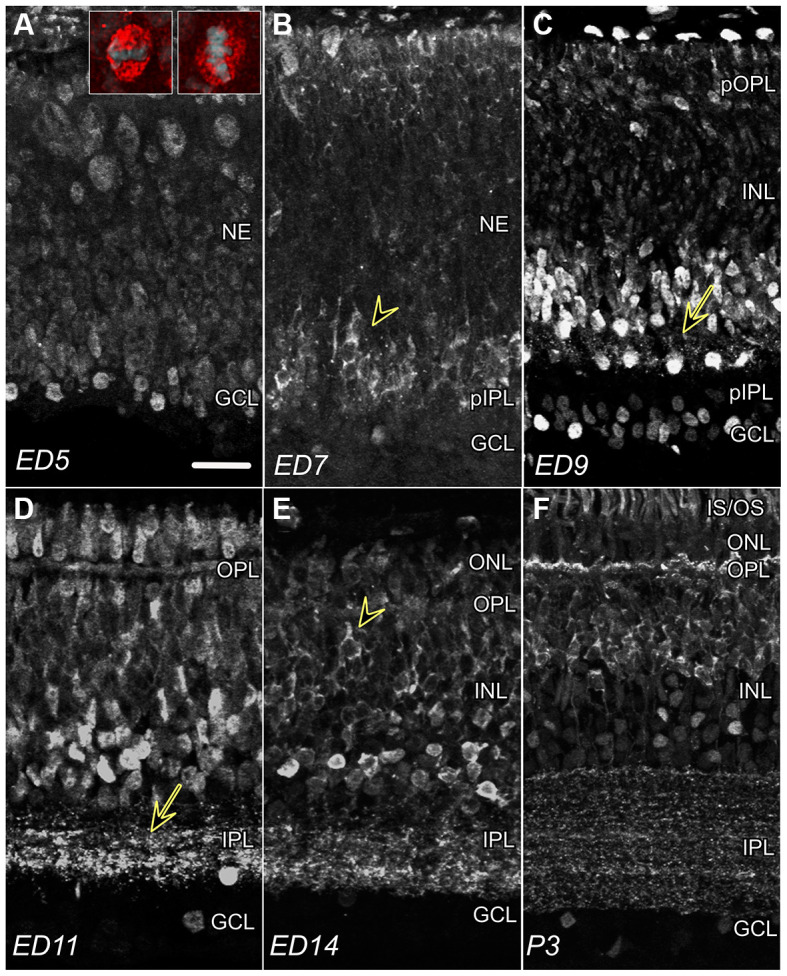
CTBP2 and RIBEYE expression in the developing chick retina. **(A)** Nuclear CTBP2 staining at ED5 visualized at opposite poles of the retina (vitreal and ventricular surfaces). Larger mitotic cells near the ventricular surface (red) are vertically or horizontally oriented evident by DAPI+ chromatin (inset panel; grey), whereas smaller post-mitotic nuclei line the vitreal surface. **(B)** CTBP2 with perinuclear and cytoplasmic (arrowhead) staining at ED7. **(C)** CTBP2 in different cell layers at ED9 including punctate staining near amacrine bipolar cell junctions (yellow arrow). **(D)** Nuclear and synaptic CTBP2 staining at ED11 with increasing cytoplasmic expression in bipolar cells. The separation between nuclear and cytoplasmic/synaptic staining at ED14 and P3 **(E,F)**. NE, neuroepithelium; pIPL, presumptive inner plexiform layer; GCL, ganglion cell layer; INL, inner nuclear layer; OPL, outer plexiform layer; ONL, outer nuclear layer; IS/OS, inner and outer segments. Scale bar = 20 μm.

**Figure 2:**
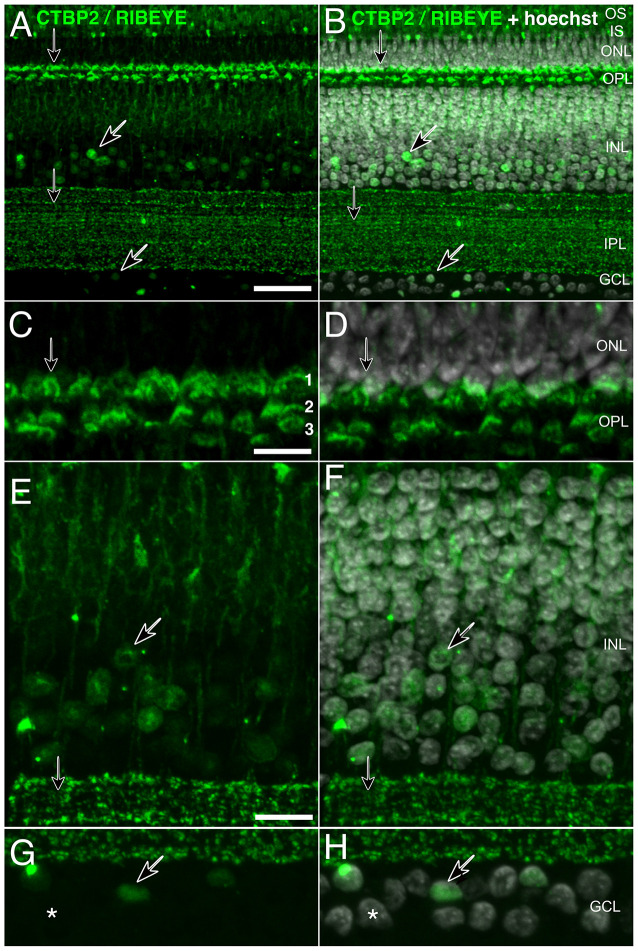
High-resolution staining of CTBP2 and RIBEYE in the mature chick retina. Cryosectioned chicken retinas at **(A,B)** P3 exhibiting CTBP2 expression in the INL and GCL (diagonal arrows) as well as the IPL and OPL (vertical arrows). **(B,D,F,H)** Sections were counterstained with Hoechst for cell contrast. **(C,D)** A tri-laminar synaptic structure representing the PR projections terminating at synaptic sublayers 1–3. **(E,F)** CTBP2 positive nuclei in the amacrine cell layer. **(G,H)** Scattered nuclei in the GCL. Scale bars = 25 μm in **(A)**, 5 μm in **(C,E,G)**.

Chick retinas were fixed in 4% paraformaldehyde (PFA) in 0.1 M phosphate buffer and 5% sucrose for 25 min, then sequentially immersed in 6.75% and 12.5% sucrose in PBS for 1 h each, 25% sucrose-PBS overnight, 1 h in a 2:1 ratio of 25% sucrose-PBS and OCT Tissue-Tek (Ted Pella), and snap-frozen on dry ice/isopentane. Frozen sections (8 μm thick) were mounted onto Superfrost Plus slides (ThermoFisher) and incubated overnight with 1:1,000 mouse anti-CTBP2 (#612044; BD Biosciences) or 1:500 rabbit anti-GABA in PBS containing 3% normal horse serum (NHS) and 0.1% Triton X-100. Secondary antibodies were anti-mouse IgG’s (H+L) coupled to Alexafluor-488 (Invitrogen, 1:1,000) and anti-rabbit Alexfluor-647 Plus (Invitrogen, 1:2,000). 10 μg/ml 4’,6-diamidino-2-phenylindole (DAPI) was used to visualize cell nuclei, and sections were processed without primary antibodies as controls. In the retina, CTBP2 Western blots exhibit bands of 50 kDa and 130 kDa corresponding to the expected sizes for both the short (nuclear) and long (synaptic) variants, while in the brain only the short isoform was detected with an IHC pattern of synaptic labeling similar to other species (Schmitz et al., 2000; Wahlin et al., 2008). GABA antiserum labels amacrine and horizontal cells by IHC from multiple species, including chicken, as previously reported (Fischer et al., 1998). Images were acquired with a Zeiss LSM 510 Meta and a Molecular Devices ImageXpress Micro Confocal High-Content Imaging System. Adjustments in brightness and contrast were made using ImageJ (NIH^1^), Affinity Designer (Serif Ltd.) and/or Adobe Photoshop (Figures 1, 2).

### Cloning of Full-Length Chicken CTBP2/RIBEYE

E18 chick retinas were harvested, mRNA isolated using a Qiagen RNAeasy RNA extraction kit, and mRNA converted to cDNA using Superscript II reverse transcriptase (Invitrogen) with oligo-d(T). To amplify the full-length chicken RIBEYE isoform, PCR was carried out with Phusion hot start high-fidelity polymerase (New England Biolabs) with primers spanning the proximal end of the EST (DR428102) to the 3’ prime end of the conserved CTBP2 transcription factor repressor isoform shared by both isoforms. The short isoform was cloned in a similar fashion. Blunt end PCR products were cloned into pENTR/D-TOPO and Sanger sequenced.

### RNA-seq Analysis

Whole retinas were harvested from ED8 and ED18 developing chicken embryos as previously described (Langouet-Astrie et al., 2016). Human pluripotent stem cells (hPSCs) and hPSC-derived retinal organoids were harvested after 25 days (D25), D100, and D180 in culture as previously described (Wahlin et al., 2017). The retina was collected from left and right pairs of whole globe human donor eyes curated from the National Disease Research Interchange as previously described (Schumacker et al., 2020). Total RNA was extracted from these tissues using a Qiagen All-Prep Mini Kit with an on column DNaseI treatment per the manufacturer’s instructions. Total RNA samples were sent for commercial Illumina sequencing (Genewiz; South Plainfield, NJ). All submitted samples had RNA integrity number (RIN) values >8 (Langouet-Astrie et al., 2016). The quality of individual sequences was evaluated using FastQC analysis. All FASTQ files have an average per base Phred score >28 which indicates high-quality base calls. ED8 and ED18 chicken retina data were combined with ED3, ED5, and ED8 RNA-seq data from a previous study (Rodrigues et al., 2016). These two data sets were normalized using the trimmed mean of M-values (TMM) method within the edgeR package (Robinson and Oshlack, 2010). Validation of chicken RNA-seq data normalization was observed using multidimensional scaling with the edgeR package (Supplementary Figure S1). High-quality chicken and human sequence reads were aligned to the galGal6 chicken and hg38 human reference genomes respectively using the HISAT2 splice aware alignment tool (Kim et al., 2015).

### Map Assembly of Short and Long CTBP2 Isoforms

FASTQ files from chicken retina RNAseq experiments were trimmed using Trimmomatic (Galaxy Version 0.38.0) and CTBP2 reads were mapped using Bowtie2 v 2.3.2 (Langmead and Salzberg, 2012).

### scRNA-seq Analysis

Previously published single-cell RNA sequencing (scRNA-seq) data were analyzed using the Broad Single Cell Portal^2^ (Yan et al., 2020; Yamagata et al., 2021). Uniform Manifold Approximation and Projection (UMAP) plots, t-Distributed Stochastic Neighbor Embedding (tSNE) plots, and dot plots data visualization were created using online tools housed within the Broad Single Cell Portal.

### Bisulfite Pyrosequencing

Quantitative analysis of DNA methylation was measured using bisulfite pyrosequencing performed as previously described (Lee et al., 2017; Hossain et al., 2018). Briefly, bisulfite conversion was performed on 200 ng genomic DNA using the EZ DNA Methylation-Gold Kit (Zymo, Irvine, CA). Following conversion, 30μl PCR reactions were performed using 2× JumpStart Taq ReadyMix (Sigma-Aldrich, St. Louis, MO). 5′ biotinylated PCR primers were designed for the 5′ regulatory regions of the target genes using PyroMark Assay Design 2.0 software (Qiagen, Hilden, Germany). The PCR cycling conditions were 95°C for 1 min, followed by 45 cycles of 95°C for 30 s, 50–58°C for 30 s, and 72°C for 30 s, with a final extension at 72°C for 1 min on a Bio-Rad C1000 Touch Thermal Cycler (Bio-Rad, Hercules, CA). Variable PCR annealing temperatures for different primer sets are indicated in (Supplementary Table S1). Biotinylated PCR products were purified and made single-stranded to serve as a template in a pyrosequencing reaction using the PyroMark Q24 Vacuum Prep Tool (Qiagen) per the manufacturer’s instructions. A sequencing primer (0.3 μM final) was annealed to the purified single-stranded PCR product, and pyrosequencing reactions were performed using the PyroMark Q24 Pyrosequencing System (Qiagen) per the manufacturer’s instructions. Percent DNA methylation at each CpG dinucleotide in the bisulfite PCR amplicon was determined and averaged between biological replicates. Statistical significance between the two sample groups was determined using a one-tailed t-test with the significance threshold set at 0.01. All PCR and sequencing primers used in these experiments are shown in Supplementary Table S1.

### Data Availability

Chicken retina RNA sequencing datasets and adult human retina samples (PRJNA275440 and PRJNA573087 respectively) were downloaded from the SRA repository. Raw sequence reads from human retinal organoids were submitted to SRA (PRJNA754196). Chicken mRNA sequences were submitted to NCBI (MZ983546, MZ983547, and MZ983548).

## Results

### Stage-Dependent CTBP2/RIBEYE Localization in the Developing Chicken Retina

To determine CTBP2/RIBEYE protein expression patterns in the developing chicken retina, IHC was performed using antibodies raised against both the long and short CTBP2 isoforms. CTBP2 was detected throughout embryonic development from the earliest period before retinal lamination through the end of retinal differentiation (Figure 1). At embryonic day (ED) 5, prior to lamination, signals were primarily detected in nuclei with the highest signals detected in cells at opposite ends near the ventricular (top) and vitreal (bottom) surfaces (Figure 1A). Cells “born” along the ventricular surface of the neuroepithelium were larger in size, possibly reflecting their mitotic status (Figure 1A; inset). Inversely, CTBP2+ cells along the vitreal surface, where ganglion cells reside, were smaller and round. By ED7, perinuclear and cytoplasmic CTBP2/RIBEYE signals were observed in the prospective inner nuclear layer (Figure 1B; INL). By ED9, CTBP2/RIBEYE signals decorated vertically aligned processes (Figure 1C; arrow) projecting from INL neurons that based on their position appeared to be ACs. Prominent nuclear staining was also observed in both the INL and GCL. Previous studies have demonstrated that RIBEYE is a key structural component of PR ribbon synapses in the retina of many vertebrates (Schmitz et al., 2000). Synaptic localization in the IPL was first noted in parallel oriented bands of speckled puncta at ED11 (Figure 1D). At ED14, a clear distinction between nuclear AC-associated CTBP2 and cytoplasmic BC-associated CTBP2 was observed (Figure 1E). Synaptic localization in the OPL was observed much later (Figure 1F). RIBEYE staining in the IPL by ED11 and OPL by ED16 demonstrated that synapses in the inner retina developed first, a concept that is supported by our previous work (Wahlin et al., 2008). Collectively, the differences in CTBP2/RIBEYE isoform distribution observed during retinogenesis are in line with previous work in chick and other vertebrates.

### Nuclear and Synaptic Isoforms Localize to Different Cell Types Within the Retina

Ribbon synapses are specialized protein structures facilitating rapid and sustained neurotransmitter release at the active zone of PR and BC retinal neuron presynaptic complexes (Sterling and Matthews, 2005). Histochemical staining of P3 chicken retinas showed clearly separated CTBP2 short and RIBEYE long isoforms (Figure 2). The short isoform was found in nuclei of ACs and cells in the GCL (Figures 2E–H; diagonal arrows). By contrast, synaptic staining was present in the OPL where PR synapses reside and over a dozen IPL sublayers where BC synaptic ribbons interface with neurons in the GCL (Figures 2A–D; vertical arrows). High magnification images of RIBEYE staining in the OPL demonstrate that CTBP2/RIBEYE was present in all three sub-laminae of the OPL where different classes of cones and rod synapses were described to connect with BC and HC dendrites (Wahlin et al., 2010).

### Developmental Regulation of Chicken *CTBP2* Isoforms

**Figure 3:**
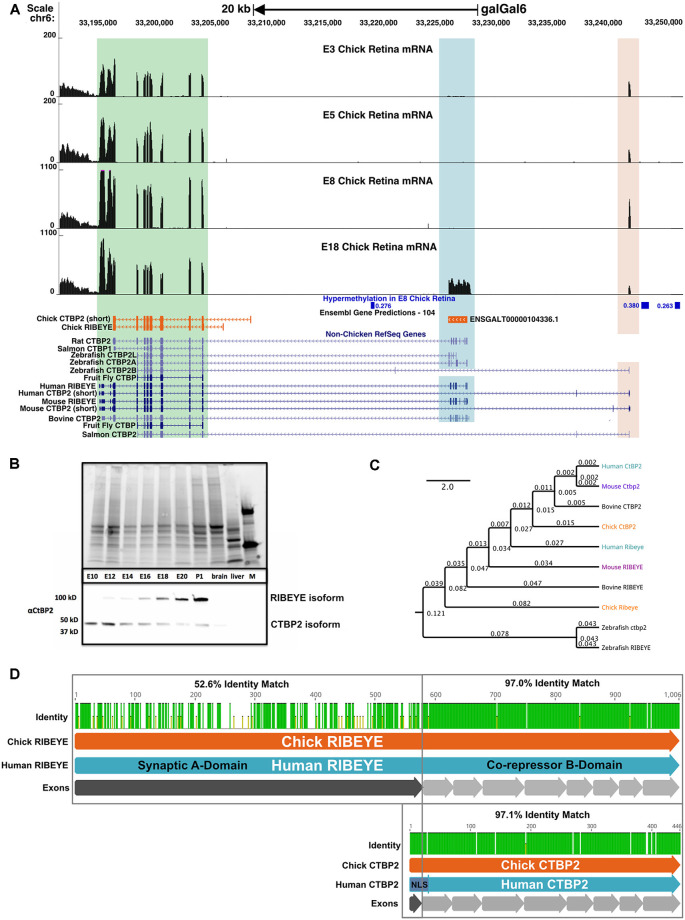
The chicken *CTBP2/RIBEYE* locus codes for developmentally regulated mRNA and protein isoforms. **(A)** UCSC Genome Browser visualization of the *CTBP2/RIBEYE* locus in the most recent chicken galGal6 genome assembly. RNA-seq alignments from ED3–18 retinas differ from the gene predictions in the Ensembl database. Multiple mRNA isoforms read from right to left (top arrow), match human (*Homo sapiens*) and mouse (*Mus musculus*) orthologous genes initiated from the TSS of the short isoform (light orange highlighted region) and the RIBEYE isoform (blue highlighted region). A green box indicates a region that is conserved between long and short isoforms. Differentially methylated regions (DMRs) previously identified between the ED8 and ED18 chicken retina are indicated in blue. Low coverage whole genome bisulfite sequencing (WGBS) analysis of the developing chicken retina identified three DMRs in the CTBP2 locus with increased DNA methylation early in retinal development. DMRs display associated *meanDiff* metrics describing the average methylation differences between E8 and ED18 retina for each respective DMR. **(B)** Coomassie-stained SDS-PAGE (top) and anti-CTBP2/RIBEYE western blot (bottom) analysis of ED10-P1 chicken retina and control (brain, liver) tissue protein lysates and a protein marker (M). Bands representing the long and short isoforms are evident in retinas. **(C)** Phylogenetic tree diagram of various CTBP2/RIBEYE sequences from human, mouse, bovine, chick, and zebrafish genomes created by global alignment with the Blosum62 cost matrix, tree-building using the Jukes-Cantor genetic distance model and the UPGMA tree build method. **(D)** Protein alignment of RIBEYE and CTBP2 isoforms with an interspecies pairwise identity highlighted for the synaptic A-and co-repressor B-domains.

To investigate gene structure and regulation of *CTBP2/RIBEYE* mRNA isoforms we performed a BLAT (Blast-like alignment tool) search with the human *RIBEYE* coding sequence using the most recent Chicken Mar. 2018 (GRCg6a/galGal6) assembly, however, this failed to identify a complete gene structure that was comparable to human and mouse orthologs (Figure 3A). Given that IHC showed both nuclear and non-nuclear proteins, it was likely that both isoforms existed, but genomic sequence information was incomplete. Although the current public annotations had only sparse EST coverage, at least one EST (DR428102) mapped to a partial *RIBEYE* sequence with homology to human *RIBEYE*. To fill in sequencing gaps, we PCR amplified and cloned a long fragment from ED18 chick embryo retina cDNA by using primers spanning the 5’ end of EST DR428102 through the 3’ prime end of the more conserved *CTBP2* isoform shared by both isoforms. BLAT alignment of this sequence fragment, mapped to chicken chromosome 6 with 99.4% identity demonstrating that the original gene annotation was incomplete. Moreover, it aligned with human (NM_022802), rat (NM_053335), mouse (NM_001170744), and zebrafish (NM_001015064) RIBEYE exons, indicating cross species conservation (Figure 3A). Separately, a recently predicted sequence (NC_052537.1; 25-Jul-2021), was derived using the Gnomon gene prediction method based on 12 mRNAs, Six ESTs, 298 long SRA reads, 13 Proteins, and RNA-seq alignments. This prediction showed a chicken genomic structure resembling the human isoforms.

To create a complete *CTBP2/RIBEYE* gene model based on actual experimental evidence, we analyzed ED8 and ED18 chicken retina RNA-seq data sets from our previous study (Langouet-Astrie et al., 2016) by aligning that to a consensus sequence derived from the predicted NC_052537.1 sequence and Sanger sequenced full length *CTBP2* and *RIBEYE* cDNA. Bowtie2 alignment for *RIBEYE*, revealed 36,127 mapped reads with 100% coverage while alignment to the short *CTBP2* isoform revealed 44,681 overlapping reads at full coverage. To display read coverage of ED3, −5, −8, and −18 retina *CTBP2* transcripts, we aligned combined RNA-seq data from two previous studies (Langouet-Astrie et al., 2016; Rodrigues et al., 2016). Since data were obtained from two different studies, we performed TMM normalization and made MDS plots to analyze sample variation (Supplementary Figure S1) which showed that normalized samples were appropriately clustered. We next aligned *CTBP2* transcripts using the HISAT2 splice-aware aligner and saw that at early time points, the short isoform was predominant as indicated by mapped reads at the short upstream exon 1 (Figure 3A; light orange highlighted region). *RIBEYE*, which is transcribed from an alternative downstream promoter, was only detected at ED18 (Figure 3A; blue highlighted region), thus confirming that RIBEYE transcripts, like the protein, are only expressed during late retinogenesis.

Epigenetic modifications, such as genomic DNA methylation and N-terminal histone tail modifications, play key roles in regulating retina-specific transcripts (Luco et al., 2011; Lee et al., 2017; Hossain et al., 2018). To investigate whether differential *CTBP2/RIBEYE* mRNA isoforms are epigenetically regulated, we analyzed previously collected low coverage whole genome bisulfite sequencing (WGBS) data identifying >43,000 differentially methylated regions (DMRs) in genomic DNA collected from ED8 and ED18 chicken retinas (Lee et al., 2017). Focused analysis of the CTBP2 locus identified three DMR regions hypermethylated in the ED8 retina relative to the ED18 retina (Figure 3A; Supplementary Table S2). These data demonstrate that DNA methylation is developmentally modulated at CTBP2 DMRs with relatively high methylation in the ED8 retina when *RIBEYE* is transcriptionally silent. This is consistent with other reports demonstrating a global inverse correlation between gene expression and hypermethylation of first introns of protein coding genes throughout vertebrate genomes (Anastasiadi et al., 2018). Inversely, at ED18 when *RIBEYE* is transcriptionally active, DNA methylation was depleted at these three DMRs (Figure 3A). These observations suggested that switching of transcriptional activation between the *CTBP2* and *RIBEYE* mRNA isoforms may rely on epigenetically regulated DNA methylation.

To correlate *CTBP2* and *RIBEYE* mRNA findings with protein expression, we carried out western blot analysis using CTBP2 antibodies (Figure 3B). A low molecular weight band, corresponding to the short CTBP2 variant, was observed at all time points analyzed. By contrast, the larger RIBEYE isoform was weakly expressed at ED12, had equal intensity to the short isoform at ED16, and was highly expressed at ED18 relative to the short isoform (Figure 3B). Phylogenetic analysis was performed to compare homology across vertebrates. Human *CTBP2* and *RIBEYE* sequences were closely related to the mouse, bovine, and chicken in descending order (Figure 3C). While mammals were closely related, zebrafish Ctbp2 and Ribeye clustered as a separate group reflecting its evolutionary divergence from birds and mammals. In terms of sequence identity, chicken and human CTBP2 proteins were highly conserved (Supplementary Figures S2, S3) with a 97.1% protein identity (Figure 3D) while RIBEYE had an identity of 70.8%. In RIBEYE, the region corresponding to the last eight exons had an identity of 96.2% while the N-terminal first exon was only 52.5% identical. Together, this data support an evolutionarily conserved pattern of expression at the mRNA and protein levels.

### Synaptic RIBEYE Is Expressed in all Chicken Photoreceptor Subtypes

**Figure 4:**
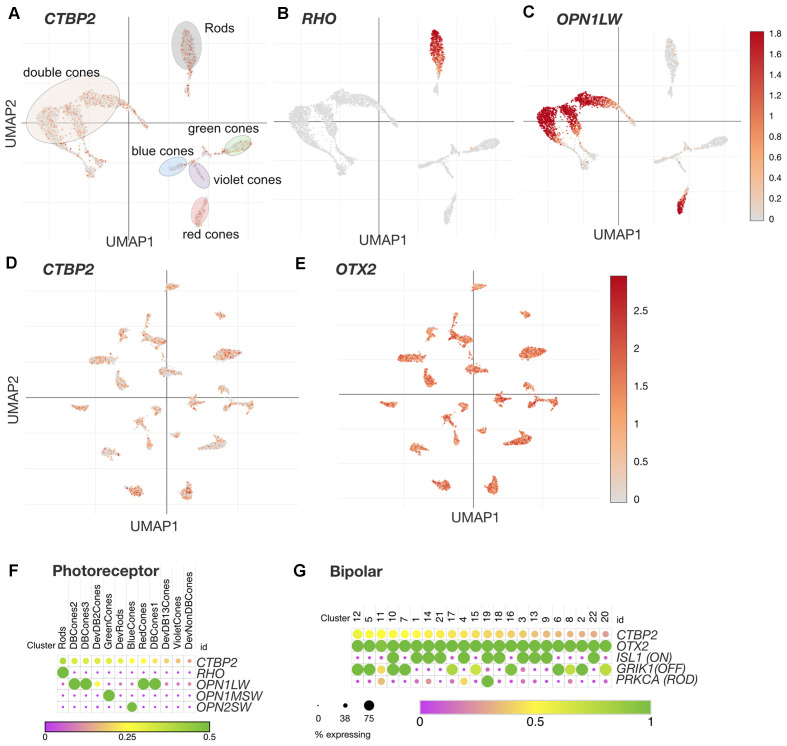
*CTBP2/RIBEYE* transcripts are expressed in all chicken PR and BC retinal neuron subtypes in the ED18 chicken retina. UMAP plots of scRNA-seq data collected from the ED18 chicken retina visualizing photoreceptor subtype cluster-specific expression of **(A)** *CTBP2/RIBEYE*, **(B)** *RHO*, and **(C)** *OPN1LW*. E18 retina UMAP plots visualizing bipolar cell subtype cluster-specific expression of **(D)** *CTBP2/RIBEYE*, and **(E)** *OTX2*. Dot plots of **(F)** Photoreceptor and **(G)** Bipolar cell expressed genes. All data were reanalyzed from a previously published study (Yamagata et al., 2021) using tools available at the Broad Single Cell Portal (https://singlecell.broadinstitute.org/single_cell). PR, photoreceptor; BC, bipolar cell.

The avian retina has a unique distribution of PR neurons that differs dramatically from that of primates and rodents. In addition to achromatic rod PRs, the chicken retina is tetrachromatic containing red, green, blue, and violet single cone (SC) PR cell types (Enright et al., 2015). The chicken retina also contains three distinct classes of double cones (DC) involved in mediating luminance detection and motion perception (Kram et al., 2010; Yamagata et al., 2021). Recent single cell RNA sequencing (scRNA-seq) analysis of the ED18 chicken retina has also demonstrated four distinct classes of immature PR precursors destined for rod, SC, and DC PR cell fates (Yamagata et al., 2021). To evaluate the cell type distribution of CTBP2 in PRs, we analyzed a scRNA-seq dataset made publicly available through the Broad Single Cell Portal (see “Materials and Methods” section; Yamagata et al., 2021). UMAP and dot plot analysis revealed that CTBP2 was present in all single cone, double cone, and rod photoreceptor cell clusters (Figures 4A,F). To demonstrate the cell-type specificity of these clusters, we inspected *RHO* and *OPN1LW* transcripts and saw that they were present only in rod and double cone/red cone PR clusters respectively (Figures 4B,C,F). Combined with histochemical evidence that all PR subtypes express the synaptic RIBEYE isoform, as opposed to the short isoform, it is likely that RIBEYE is expressed in all PR types.

### Synaptic RIBEYE Is Expressed in all Chicken Bipolar Subtypes

Based on scRNA-seq analysis of cell type-specific markers in the ED18 chicken retina, there are 22 classes of BC neurons (Yamagata et al., 2021). UMAP analysis of these showed that nearly all BC clusters expressed *OTX2* (Figure 4E), however, not all *OTX2+* cells were *CTBP2+* (Figure 4D). Dot plot analysis of *OTX2* and *CTBP2* showed a similar trend with CTBP2 that was detected in many, but not all BCs (Figure 4G). Due to the somewhat low coverage of scRNA-seq, we cannot rule out the possibility that more BCs expressed CTBP2. Given the strong immunostaining for CTBP2 in presynaptic ribbon complexes in the IPL, and absent nuclear staining, the *CTBP2* expression observed is likely due to the long *RIBEYE* isoform.

### Nuclear CTBP2 Is Preferentially Expressed in GABAergic Amacrine Cells in the Mature Chicken Retina

**Figure 5:**
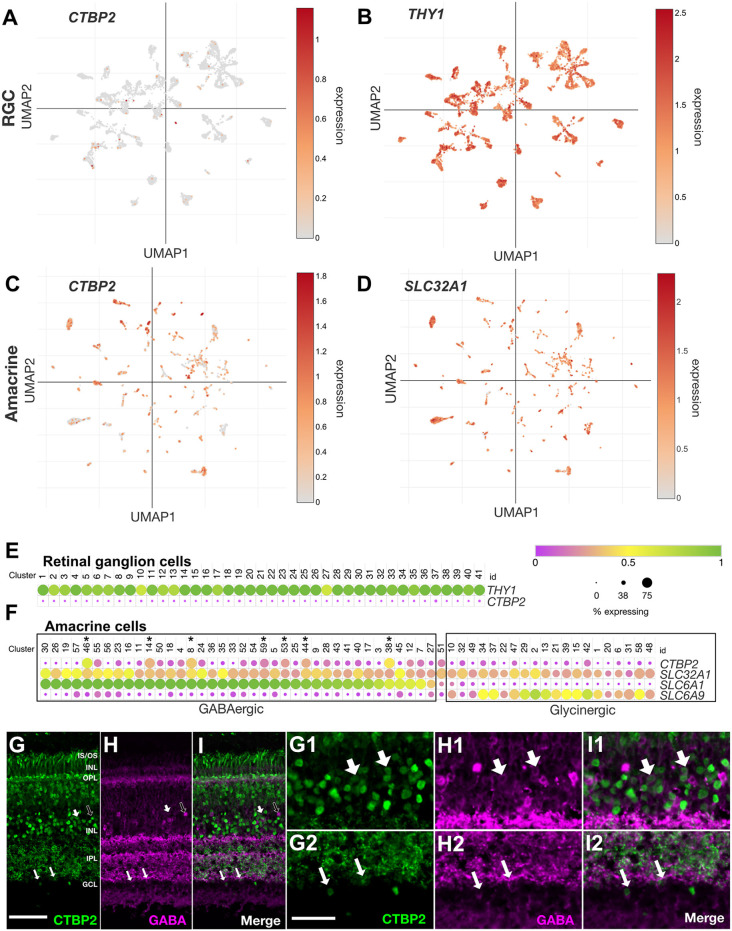
*CTBP2* transcripts are preferentially expressed in GABAergic amacrine cells in the ED18 chick retina. UMAP plots of scRNA-seq data collected from the ED16 and ED18 chicken retina visualizing RGC and amacrine cell subtype-specific expression respectively of **(A,C)** *CTBP2*, **(B)** *THY1*, and **(D)** *SLC32A1*. Dot plots of scRNA-seq data collected from **(E)** the ED18 chicken retina visualizing RGC coexpression of *CTBP2* and *THY1* and **(F)** amacrine cell subtype-specific coexpression of *CTBP2, SLC32A1*, *SLC6A1*, and *SCL6A9*. All data were reanalyzed from a previously published study (Yamagata et al., 2021) using tools from the Broad Single Cell Portal (https://singlecell.broadinstitute.org/single_cell). Immunohistochemical staining of ED18 chicken retina with **(G)** CTBP2, **(H)** GABA, and **(I)** a merged image of CTBP2/GABA. **(G1–I2)** Higher magnification areas showing the overlap in **(G1,H1,I1)** amacrine cells of the INL and **(G2,H2,I2)** displaced amacrine cells of the ganglion cell layer are indicated by large and small arrows respectively. Dark arrows in **(G–I)** indicate GABA positive cells that are CTBP2 negative. Scale bar **(G–I)** = 50 μm; scale bar **(G1–H2)** = 25 μm.

Histochemical and western blot data demonstrated that CTBP2 was abundant in early prelaminated and late-stage differentiated retinas (Figures 1, 3). At late stages, nuclear CTBP2 was limited to cells in the INL, and RGC layers (Figure 2). To determine whether CTBP2 labeled RGCs, UMAP analysis of the *THY1* transcript was used to visualize *CTBP2* expression in the 41 chicken RGC subtype clusters (Figure 5B; Yamagata et al., 2021). UMAP and dot plot analysis showed that *CTBP2* did not coexpress with *THY1+* RGC clusters (Figures 5A,E). Thus, while IHC detection of CTBP2 was + in the RGC layer, a more likely scenario is that CTBP2 was expressed in displaced ACs residing there. This contrasts with other vertebrate species including human, non-human primate and mouse which showed detectable levels of *CTBP2* in some RGCs (Supplementary Figure S4). Dot plot analysis also confirmed that *CTBP2/RIBEYE* was not significantly expressed in HCs or Müller glia (MG) in the ED18 chick retina (not shown).

ACs are the most heterogeneous class of neurons in the vertebrate retina. UMAP visualization of the AC-associated vesicular inhibitory transporter *SLC32A1*, which transports both GABA and glycine into synaptic vesicles, allowed us to visualize single cells assigned to all 59 chicken AC subtype clusters (Figure 5D; Yamagata et al., 2021). While all subtypes expressed *SLC32A1*, *CTBP2* was detected in some but not all AC clusters (Figure 5C). To explore this further, we compared the expression of GABA- (*SLC6A1*) and glycine (*SLC6A9*)-specific transporters. Cells expressing high levels of *SLC6A1* had low levels of *SLC6A9* and *vice versa* demonstrating the mutual exclusivity of these AC subtype-selective markers (Figure 5F). Interestingly, the majority of *CTBP2*+ AC subtypes were GABAergic while glycinergic ACs were largely devoid of *CTBP2* (Supplementary Figures S4F–I). Despite this subtype-specificity, only a handful of GABAergic ACs expressed significant levels of *CTBP2*. For example, *CTBP2* was not detected in GABAergic starburst ACs co-expressing *CHAT* and *GBX2* (Supplementary Figures S4F–I). A comparison between human, mouse, chicken, and non-human primates revealed that these observations were evolutionarily conserved. Although IHC can often help to correlate protein expression to specific cell types, scRNAseq was better suited for the current study since antibodies that label GABA or glycine synthetic machinery and have a nuclear localization are unavailable, thus is difficult to correlate CTBP2 and GABA or glycine synthesis. Using antibodies directed against GABA (Figures 5H,I), as opposed to the enzymes that make it, we showed that some areas of overlap, between CTBP2 and GABA existed, albeit in different subcellular compartments; CTBP2 was present in the nucleus while GABA was highly enriched in the synaptic layers (Figures 5G–I). At higher magnification, we detected colocalized signals for CTBP2 and GABA in amacrine cells in the INL as well as displaced amacrine cells in the GCL (Figures 5G1–I2). Since it is possible that GABA was taken up by nearby cells, we cannot rule out the possibility that some GABA signals were made in non-amacrine cells. Detailed gene expression by scRNAseq allowed us to fill in the apparent gaps in IHC data. Collectively, these findings demonstrated that nuclear *CTBP2* was expressed in some but not all subclasses of GABAergic ACs in the INL and GCL. By contrast, it was not detected in glycinergic ACs, RGCs, HCs, or MG by scRNAseq.

### *CTBP2 and RIBEYE* Display Similar Expression Patterns in Mature Human and Chicken Retinas

**Figure 6:**
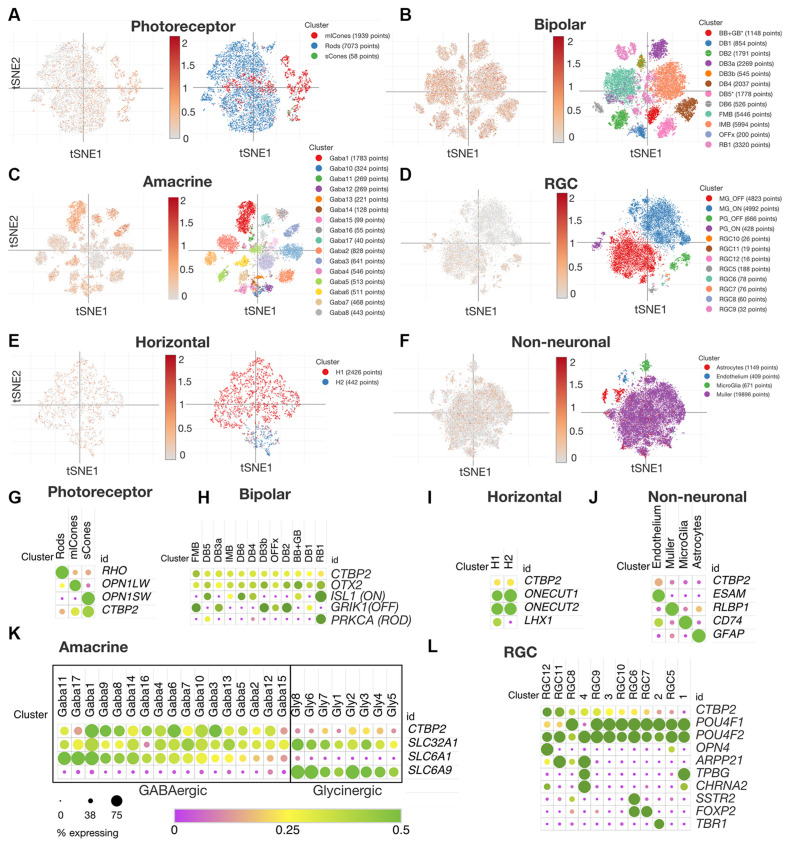
*CTBP2* expression in single cells within the adult human retina. tSNE expression analysis for adult human **(A)** photoreceptors, **(B)** bipolar cells, **(C)** amacrine cells, **(D)** RGCs, **(E)** horizontal cells, and **(F)** non-neuronal cells. Dot plot coexpression analysis of **(G)** photoreceptors, **(H)** bipolar cells, **(I)** horizontal, **(J)** non-neuronal, **(K)** amacrine, and **(L)** RGCs. All data were reanalyzed from a previous study (Yan et al., 2020) with the Broad Single Cell Portal (https://singlecell.broadinstitute.org/single_cell). RGCs, retinal ganglion cells.

Though the chicken retina is an excellent model for cone dominant vision, there are many anatomical and molecular differences that have arisen since the ~310 million years that chicken and mammalian genomes diverged (International Chicken Genome Sequencing Consortium, 2004). To compare *CTBP2/RIBEYE* expression patterns in mature chicken and human retinas, we data mined a publicly available human retina scRNA-seq dataset (Yan et al., 2020). Using t-distributed stochastic neighbor embedding (tSNE) plots we were able to visualize *CTBP2* in PR, BC, AC, RGC, HC and non-neuronal subtype clusters in the adult human retina (Figures 6A–F). Using dot plot analysis, we observed that all human PR and BC subtypes expressed *CTBP2* (Figures 6G,H). This is correlated with chicken IHC that showed *RIBEYE* protein at their synaptic terminals (Figure 2) and scRNA-seq that detected transcripts in PR and BCs (Figure 4). In addition, *CTBP2* was detected in small numbers of HCs and non-neuronal cells (e.g., endothelium) (Figures 6I,J). In chick, *CTBP2* was detected in GABAergic ACs, but not glycinergic ACs, RGCs, HCs or MG (Figure 5, Supplementary Figure S4). In human AC subtypes, *CTBP2* clustered closely with the GABAergic transporter *SLC6A1*, rather than the glycinergic transporter *SLC6A9*, like in the chicken retina (Figure 6K). A notable difference was that while chicken RGCs were largely *CTBP2* negative (Figure 5E), human RGCs did express *CTBP2* (Figure 6L). Interestingly, *CTBP2* was also detected in mouse and non-human primate RGCs, but not in zebrafish suggesting evolutionary divergence (Supplementary Figure S4). Collectively, this data demonstrated that RIBEYE and CTBP2 was expressed in PRs, BCs, and ACs in a highly conserved fashion between chickens and humans, however the human retina may have expanded usage of these gene isoforms in other retinal neurons.

### *CTBP2/RIBEYE* in Human Stem Cell-Derived Retinal Organoids Shows Conserved Epigenetic Regulation

**Figure 7:**
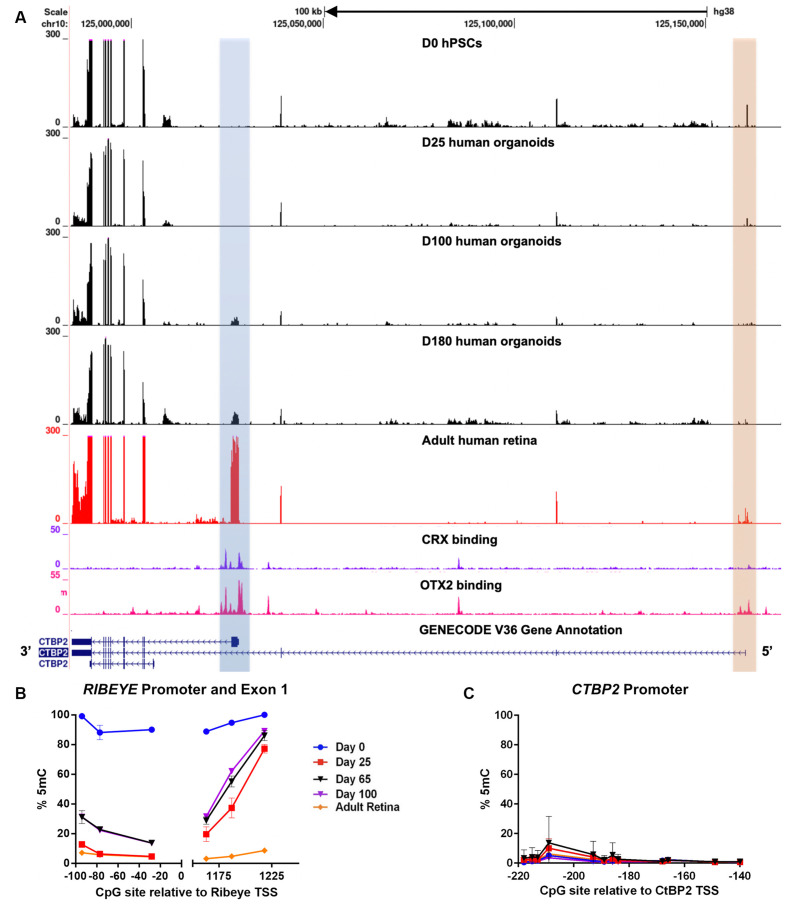
RNA-seq and DNA methylation analysis of developing human retinal neurons. **(A)** UCSC genome browser output of the *CTBP2/RIBEYE* locus aligned with RNA-seq data from pluripotent stem cells differentiated into human retinal organoids (days 0–180), as well as RNA-seq and CRX ChIP-seq data from adult human retinas. **(B)** Quantitative DNA methylation analysis of the *RIBEYE* promoter and exon 1, and **(C)** the *CTBP2* promoter.

After establishing that *CTBP2/RIBEYE* expression was similar in chicken and human retinas we next sought to evaluate the regulation of *CTBP2* isoforms during human retinogenesis. To accomplish this, we employed a human pluripotent stem cell (hPSC)-derived 3D retinal organoid system that mimics fetal human retinal development and expresses CTBP2/RIBEYE proteins by 160 days (Wahlin et al., 2017). Using RNA-seq analysis with a developmental time course of organoids, we searched for *CTBP2* short and long isoforms and saw that the primary isoform at day 0 and 25 organoids was the short form (Figure 7A; orange box). It was not until day 100 that *RIBEYE* began to be detected (Figure 7A; blue box). This well-defined peak was also present in the adult human retina. These mRNA signatures demonstrated conserved transcriptional regulation of *CTBP2/RIBEYE* between chickens and humans.

Interactions between transcription factors and genomic cis-regulatory elements (CREs) are critical for regulating the timing, levels, and cell type specificity of gene expression in the retina (Corbo et al., 2010; Cherry et al., 2020). The K50 homeodomain transcription factors OTX2 and CRX have been previously shown to act on a subset of human retinal CREs to orchestrate complex transcriptional regulation (Cherry et al., 2020). To determine if the *CTBP2/RIBEYE* locus was regulated by K50 transcription factors, we analyzed OTX2 and CRX ChIP-seq alignments from a retrospective study (Cherry et al., 2020). ChIP-seq data from adult human retinas demonstrated that OTX2 had multiple binding sites at the *CTBP2* locus, including the proximal promoter regions of both mRNA isoforms (Figure 7A). CRX binding was also observed at the proximal *RIBEYE* isoform TSS, however, no binding was detected near the distal short *CTBP2* isoform TSS (Figure 7A). This data implies differential transcriptional regulation of human *CTBP2/RIBEYE* by retinal transcription factor CREs.

CRX binds to six base pair consensus motifs embedded in CRE sequences in and adjacent to PR-specific genes (Corbo et al., 2010). CRX binding motifs, however, are present in millions of copies within mammalian genomes, most of which are spurious nonfunctional motifs (White et al., 2013). In addition to specific binding motifs, previous studies demonstrated that CRX binding affinity is partially determined by other sequence features including local GC content and epigenetic modifications adjacent to CRX motifs (White et al., 2013; Hossain et al., 2018). We previously demonstrated that human retina-specific gene expression is associated with differential levels of DNA methylation in and adjacent to CRX binding sites near TSSs (Hossain et al., 2018). Though the chicken genome does not encode a CRX ortholog, we did observe developmentally regulated differentially methylated regions (DMRs) proximal to the *RIBEYE* mRNA isoform TSS (Figure 3A). We performed similar analyses at the human *CTBP2/RIBEYE* locus using bisulfite pyrosequencing (BSPY) on DNA isolated from hPSC-derived retinal organoids and showed that functional CRX binding sites adjacent to the *RIBEYE* TSS were initially methylated in undifferentiated stem cells when it is transcriptionally silent (Figure 7B). Over time, CRX binding sites at the *RIBEYE* locus became demethylated coinciding with its transcriptional activation (Figure 7B). This site was also demethylated in adult human donor retinas where *RIBEYE* is abundantly transcribed. Unlike *RIBEYE* which demonstrates temporally regulated methylation and transcription, the short *CTBP2* isoform TSS was demethylated and expressed in all samples analyzed (Figure 7C). This suggested that switching between CRX-independent *CTBP2* isoform and CRX-dependent *RIBEYE* isoform expression was epigenetically regulated by DNA methylation. Overall, CTBP2 proteins existed as synaptic and nuclear isoforms, their expression was transcriptionally conserved between humans, and chicken and it showed similarities in epigenetic regulation.

## Discussion

The vertebrate retina is a heterogeneous collection of diverse neurons and neuronal support cells that can be molecularly characterized by their unique transcriptional profiles. Complex regulation of gene expression during retinal development allows for the multifaceted utility of individual genomic loci. In this study, we used IHC and sequencing analysis to demonstrate the dynamic regulation of the multiuse *CTBP2/RIBEYE* locus in the developing vertebrate retina. Our findings can be summarized as follows: (1) The chicken genome encodes a functional *CTBP2* gene with similar structural features, transcriptional regulation, and isoform production as those found in mammalian genomes. (2) Chicken CTBP2 and RIBEYE proteins were observed in nuclei and synapses respectively of retinal neurons. (3) The mature chicken retina preferentially expressed the CTBP2 transcriptional corepressor in GABAergic ACs within the INL and GCL while the RIBEYE protein was observed in PR and BC ribbon synapse complexes residing in the OPL and IPL. (4) Differential DNA methylation during development provided epigenetic transcriptional regulation of the *CTBP2/RIBEYE* locus.

Expression of RIBEYE in cells other than PRs and BCs has not been reported in chick or other species and for the most part, this was true in our study. An exception was the cytoplasmic CTBP2+ signals in cells located in the inner aspect of the INL. While numerous AC subtype clusters, including SLC6A1+ GABAergic ACs coexpressed CTBP2, this approach lacks the depth of coverage to accurately assess isoform specificity. One possibility is that CTBP2+ puncta might originate from BC terminals that are just beginning to form. In the mouse retina, BC axons and dendrites originate from neuroepithelial-like processes spanning the width of the retina (Morgan et al., 2006). As they mature, their nuclei and cell soma migrate, and processes retract. Since BCs are generated by ED7 and axonal extensions project through the AC layer (Gallego, 1986) this could explain the unusual CTBP2 staining pattern.

Using a novel hPSC-derived retinal organoid cell culture system, we demonstrate that transcriptional regulation of the *CTBP2/RIBEYE* multiuse locus is similarly orchestrated during chick and human development (Figures 3A, 7A). Early and persistent expression of the shorter nuclear CTBP2 isoform suggests a general role as a transcriptional coregulator in diverse retinal precursors and differentiated retinal neurons. The longer synaptic RIBEYE isoform is expressed later in development as ribbon synapse complexes begin to form. Gene expression networks associated with developing synaptic ribbons have been previously shown to be dependent on the K50 homeodomain transcription factor CRX (Assawachananont et al., 2018). Using data from a previous human retina ChIP-seq study, we show that cis-regulatory elements (CREs) adjacent to the *RIBEYE* TSS but not the *CTBP2* TSS, are bound by CRX (Figure 7A). Epigenetic regulation of the *RIBEYE* isoform likely plays a prominent role in its temporal expression. Genomic regions adjacent to the *RIBEYE* TSS are densely methylated at early time points in both chick and human retinal development but become demethylated as development progresses (Figures 3A, 7A). The binding of CRX to CREs has been shown to be dependent on local sequence features other than DNA base pair grammar (Walker et al., 2010; Hossain et al., 2018). Collectively, these data suggest a regulatory mechanism for CRX binding affinity to *RIBEYE* CREs that is modulated in part by DNA methylation. Further investigation is required to determine the biochemical effect of differential DNA methylation on K50 homeodomain transcription factor binding affinity to target CREs at the *RIBEYE* locus as well as other PR-specific genes.

The Broad Single Cell Portal (Yamagata et al., 2021) is a searchable atlas of genes expressed in the chicken retina that allowed us to identify *CTBP2* expression in multiple cell types including PRs, BCs, and ACs. Combined with IHC staining, scRNA-seq provided cell type specific resolution of gene transcripts. While RIBEYE is well known for its role in glutamate release from PR and BC terminals, the role of the shorter *CTBP2* isoform has not been extensively studied in the eye but rather from cancer and stem cell studies (Chew and Gallo, 2009; Kim et al., 2017; Chen et al., 2020; He et al., 2021). CTBP2 itself does not directly bind to DNA but instead interacts with other proteins including COREST, CTBP1, PC2, EHMT, FHL3, G9A, HDAC1, HDAC2, HDM2, KLF3, NRIP, SOX6, ZEB1, ZFHX1, and ZNF217 among others, that form a complex that interacts with DNA. While we have little mechanistic data on the CTBP2 complex in the retina, in the inner ear CTBP2 and HDACs are recruited by SOX proteins, which regulate gene expression through transcriptional repression (Chew and Gallo, 2009). Given the role of SOX proteins in the retina, a similar interaction may occur here too. During neural development, *CTBP2* seems to participate in cell proliferation and is robustly expressed in ventricular zone cells where active cell division occurs (Karaca et al., 2020). In fact, *CTBP2* null mice exhibit delayed development resulting in thinning of the neural epithelium, including the retina, and eventual degeneration (Hildebrand and Soriano, 2002). In terms of CTBP2 expression in the mature retina, we observed the short *CTBP2* isoform in GABAergic ACs, which may indicate a connection between CTBP2 transcriptional regulation of genes specifying inhibitory neurotransmitter activity. Lastly, while CTBP2 is typically recognized as a repressor, it can also act as a coactivator when bound to the retinoic acid receptor/retinoid X receptor (Bajpe et al., 2013), which is expressed in PRs. Given the presence of the cellular retinoic acid binding protein (CRABP) in amacrine and bipolar cells of the chick eye, CTBP2 and retinoic acid receptors may coordinate to regulate amacrine and/or bipolar cell function (Fischer et al., 1999). The role of CTBP2 in retinal development and homeostasis thus remains largely unknown and additional work is necessary to understand the binding partners and gene networks it regulates at different stages of development.

The conspicuous downregulation of nuclear CTBP2 protein in retinal neurons prior to synaptogenesis and a simultaneous shift in synaptic protein expression is a fascinating example of a single gene producing multiple isoforms with dramatically different functions. Our findings demonstrate that epigenetic regulation of K50 homeodomain transcription factor affinity may play a prominent role in modulating transcription at this multiuse locus. Although the current data fill in critical gaps in our knowledge of CTBP2 gene structure and transcriptional regulation in the chicken retina, many questions remain including how alternative promoters are engaged in developing cells. Transcription factors and CREs control dynamic genetic programs that enable neural development, but it is not known when, where, or how these engage or repress gene expression. Future efforts to elucidate the CTBP2 complex could lead to a greater understanding of its role in retinal development and homeostasis.

## Data Availability Statement

Chicken retina RNA sequencing datasets (PRJNA352493, PRJNA275440) and adult human retina RNA sequencing datasets (PRJNA573087) were downloaded from the SRA repository. The names of the repository/repositories and accession number(s) can be found below: https://www.ncbi.nlm.nih.gov/, SRR4929902, SRR4929903, SRR4929904, SRR1804237, SRR1804238, SRR1804239, SRR1804240, PRJNA573087, and PRJNA754196.

## Ethics Statement

The animal study was reviewed and approved by James Madison University IACUC committee.

## Author Contributions

EG: collection and assembly of data, data analysis and interpretation, manuscript writing, and final approval of manuscript. DA: collection and/or assembly of data, data analysis and interpretation, manuscript writing, and final approval of manuscript. CC and SS: collection and assembly of data, final approval of manuscript. KW-B, NJ, and ZW: collection and/or assembly of data, final approval of manuscript. MJ: collection of organoids and final approval of manuscript. DZ: data analysis and final approval of manuscript. RE and KW: conception and design, collection and/or assembly of data, data analysis and interpretation, manuscript writing, and final approval of manuscript. All authors contributed to the article and approved the submitted version.

## Conflict of Interest

The authors declare that the research was conducted in the absence of any commercial or financial relationships that could be construed as a potential conflict of interest.

## Publisher’s Note

All claims expressed in this article are solely those of the authors and do not necessarily represent those of their affiliated organizations, or those of the publisher, the editors and the reviewers. Any product that may be evaluated in this article, or claim that may be made by its manufacturer, is not guaranteed or endorsed by the publisher.

## Footnotes

^1^ http://rsb.info.nih.gov/ij/

^2^ https://singlecell.broadinstitute.org/single_cell
