# Supplementary material for: Temporal and Isoform-Specific Expression of CTBP2 Is Evolutionarily Conserved Between the Developing Chick and Human Retina
Source: Front Mol Neurosci. 2022 Jan 13;14:773356. doi: 10.3389/fnmol.2021.773356 (PMC8793361; doi:10.3389/fnmol.2021.773356)
Supplement: Supplementary file 1 [file Data_Sheet_1.PDF]

Supplemental Materials

Primer Name	Sequence (5′-3′)	Region Analyzed	Amplicon Size (bp)	Annealing Temp (°C)	Application
hCTBP2-early-F1	GGGGTAGGGTTAGGGA GTGT	CTBP2 isoform promoter	128	54	BS PCR
hCTBP2-early-R1	Bio-CCCCTTCAAATACTCCT CACTACAAAT	CTBP2 isoform promoter	128	54	BS PCR
hCTBP2-early-seq1	GTTTGTGTGTATTTT	CTBP2 isoform promoter	N/A	N/A	Pyro sequencing
hCTBP2-early-seq2	GGGTTAGGGAGTGTT	CTBP2 isoform promoter	N/A	N/A	Pyro sequencing
hCTBP2-late-pro-F1	TGGGAGGGTTGGATAG AGTAAGT	RIBEYE isoform promoter	143	54	BS PCR
hCTBP2-late-pro-R1	Bio-CCCCCTTCCTAACTAAT ATACTCACAT	RIBEYE isoform promoter	143	54	BS PCR
hCTBP2-late-pro-seq1	GGTTGGATAGAGTAAG TTAT	RIBEYE isoform promoter	N/A	N/A	Pyro sequencing
hCTBP2-late-ex1-F1	GTTTTTTTTTAGGGATT GAGTTGTAAGGA	RIBEYE isoform exon 1	159	52	BS PCR
hCTBP2-late-ex1-R1	Bio-AAACAACCAACTAAAA ATTTTCTATTTC	RIBEYE isoform exon 1	159	52	BS PCR
hCTBP2-late-ex1-seq1	GAGTTGTAAGGAATAG ATTT	Ribeye isoform exon 1	N/A	N/A	Pyro sequencing

Table S1. PCR and sequencing primers used in this study. 5’ biotinylation modification (Bio) is indicated on reverse pyrosequencing primers.

chr	start	end	MeanDiff	areaStat	tstat.sd
Chr6	33218263	33218579	0.276	14.2838502	0.09668239
Chr6	33245301	33246009	0.380	59.1333174	0.08986108
Chr6	33249989	33250421	0.263	8.76293565	0.09013309

Table S2. Quantitative analysis of 3 DNA hypermethylated methylated regions (DMR) in ED8 chicken retinas from low coverage whole genome bisulfite sequencing (WGBS). WGBS was conducted in triplicate and is quantitative with associated statistics. The 1st 3 columns show the genome coordinates. MeanDiff represents the average methylation difference in a DMR region, The areaStat=area under the t-statistic curve is used for ranking the DMRs, and tstat.sd=standard deviation of the t.stat shows variation in the 3 replicates.

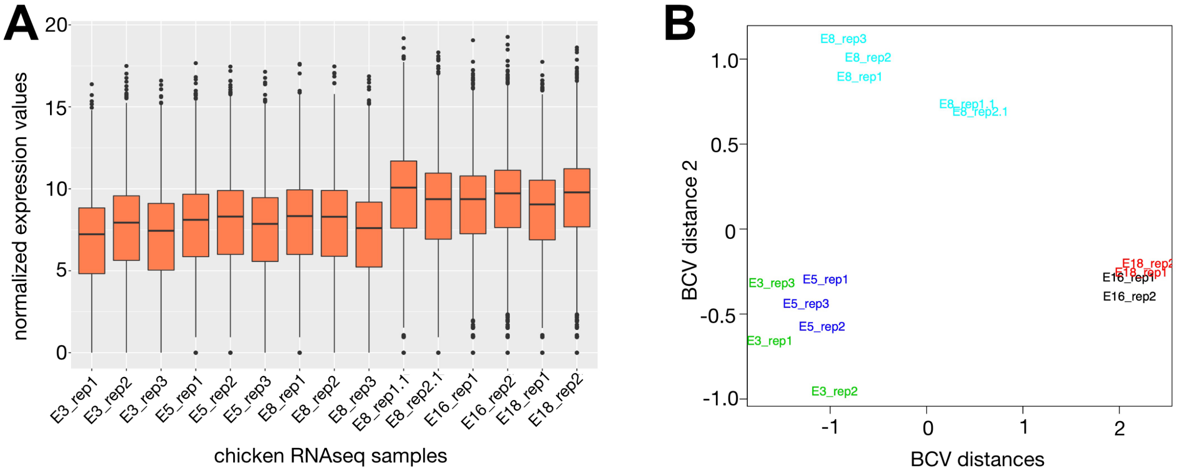

Figure S1. TMM normalization and MDS of combined ED3-ED18 chicken RNA-seq data sets. (A) A plot representing chicken retina RNA-seq data sets from two independent studies combined and normalized using the trimmed mean of M-values (TMM) functionality within the edgeR package. (B) An MDS plot output from EdgeR showing clustering among E3, E5, E8, 16 and 18 chick retinas.

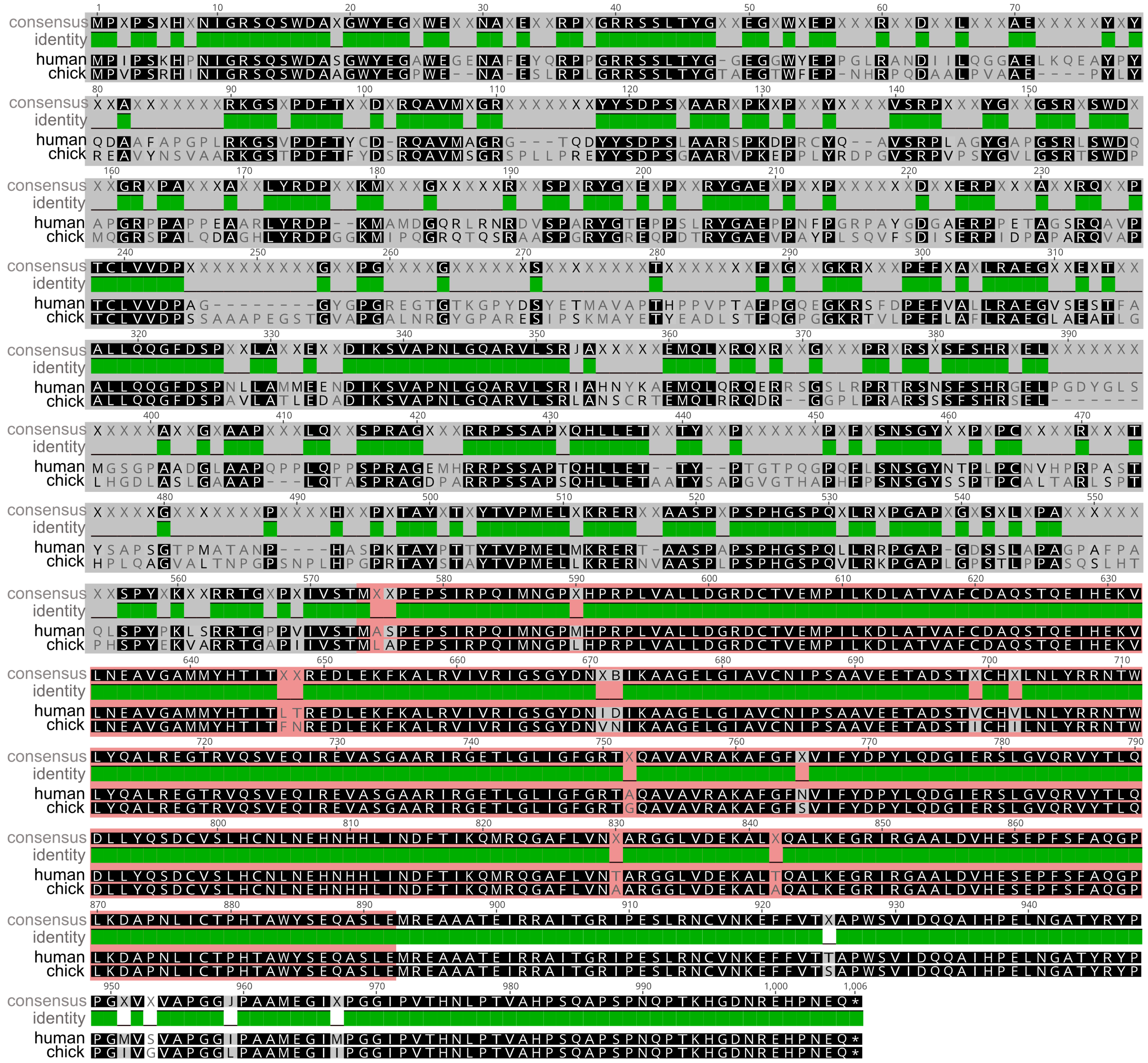

Figure S2. Alignment of human and chick *RIBEYE*. A pairwise alignment between the full-length chicken and human *RIBEYE* sequences. Identity between amino acids is highlighted with a black background and green bar above. The RIBEYE domain is shown with a grey background, while the conserved repressor domain is shown in red.

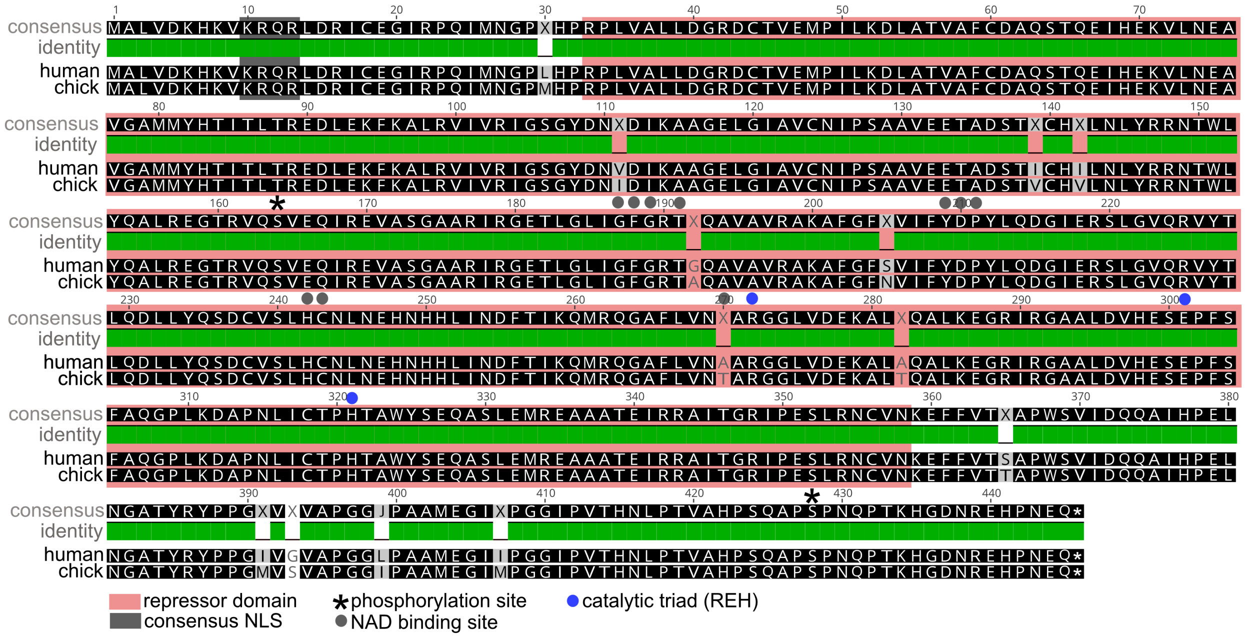

Figure S3. Alignment of human and chick *CTBP2*. A pairwise alignment between the full-length chicken and human *CTBP2* sequences. Identity between amino acids is highlighted with a black background and green bar above. The conserved transcriptional repressor domain is shown in red. Putative phosphorylation sites are indicated by *, NAD binding sites are indicated with a black dot, and the catalytic triad indicated as blue dots.

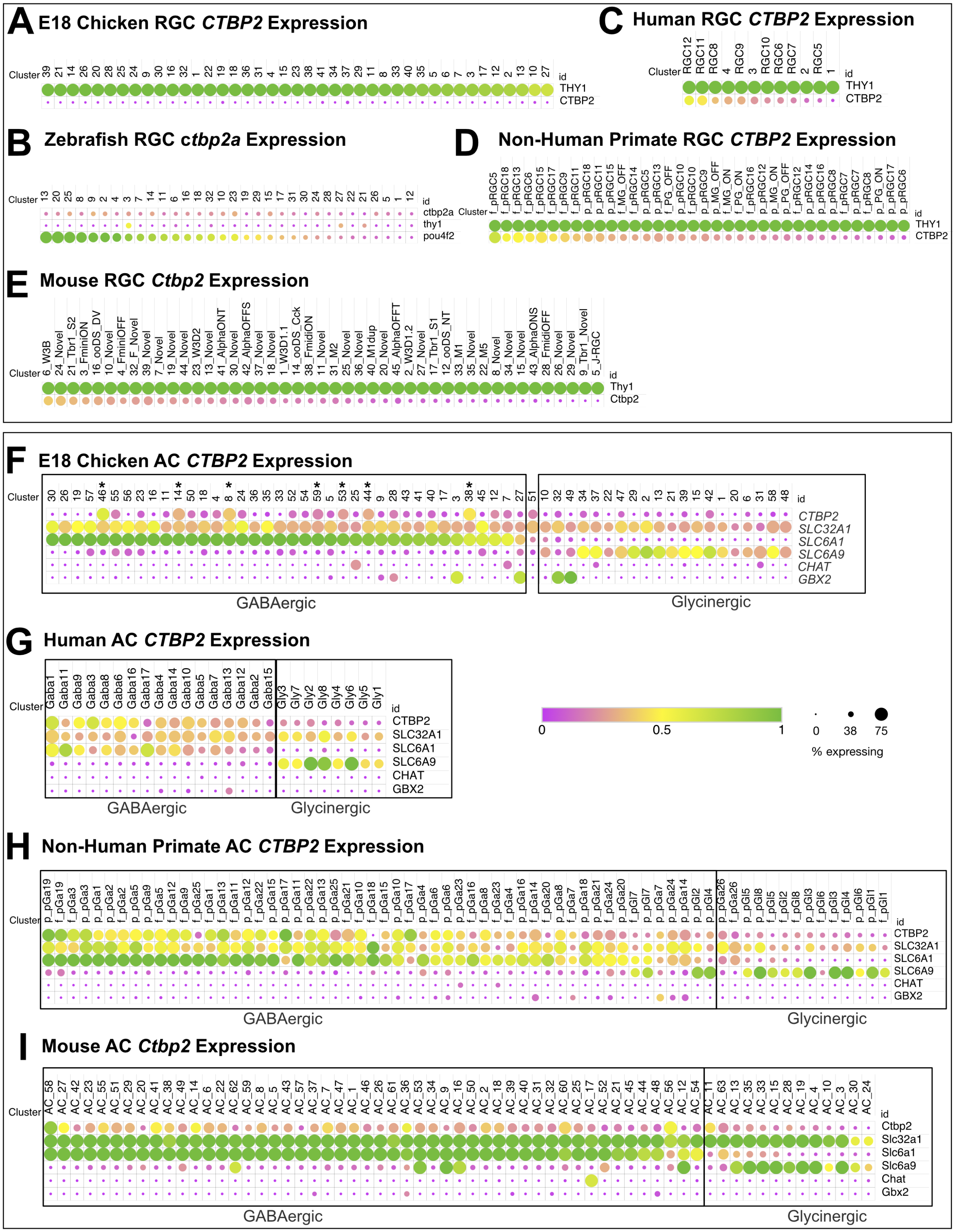

Figure S4. Multispecies comparison of *CTBP2* expression in RGCs and amacrine cells. Dot plots of scRNA-seq data collected from (A) the ED18 chicken, (B) zebrafish (C) human, (D) non-human primate and (E) mouse retina visualizing RGC co-expression of *CTBP2/RIBEYE* and *THY1* as well as amacrine cell

subtype-specific coexpression of *CTBP2/RIBEYE*, *SLC32A1*, *SLC6A1*, *SCL6A9*, *CHAT*, and *GBX2* from (F) the ED18 chicken, (G) human, (H) non-human primate and (I) mouse retina. All data were reanalyzed from previously published studies (1-4) using tools from the Broad Single Cell Portal (https://singlecell.broadinstitute.org/single_cell).
